# X-linked inhibitor of apoptosis protein (XIAP) lacking RING domain localizes to the nuclear and promotes cancer cell anchorage-independent growth by targeting the E2F1/Cyclin E axis

**DOI:** 10.18632/oncotarget.2227

**Published:** 2014-07-17

**Authors:** Zipeng Cao, Xueyong Li, Jingxia Li, Wenjing Luo, Chuanshu Huang, Jingyuan Chen

**Affiliations:** ^1^ Department of Occupational and Environmental Health and Ministry of Education Key Lab of Hazard Assessment and Control in Special Operational Environment, School of Public Health, Fourth Military Medical University, Xi’an, China; ^2^ Nelson Institute of Environmental Medicine, New York University School of Medicine, Tuxedo, NY, USA; ^3^ Department of Plastic and Burn Surgery, Tangdu Hospital, Fourth Military Medical University, Xi’an, China

**Keywords:** XIAP, RING domain, cell cycle progression, E2F1

## Abstract

The inhibitor of apoptosis protein XIAP (X-linked inhibitor of apoptosis protein) is a well-documented protein that is located in cytoplasm acting as a potent regulator of cell apoptosis. Here, we showed that expressing XIAP with RING (Really Interesting New Gene) domain deletion (XIAP^ΔRING^) in cancer cells promoted cancer cell anchorage-independent growth and G_1_/S phase transition companied with increasing *cyclin e* transcription activity and protein expression. Further studies revealed that XIAP^ΔRING^ was mainly localized in nuclear with increased binding with E2F1, whereas XIAP with BIR (Baculoviral IAP Repeat) domains deletion (XIAP^ΔBIRs^) was entirely presented in cytoplasma with losing its binding with E2F1, suggesting that RING domain was able to inhibit BIR domains nuclear localization, by which impaired BIRs binding with E2F1 in cellular nucleus in intact cells. These studies identified a new function of XIAP protein in cellular nucleus is to regulate E2F1 transcriptional activity by binding with E2F1 in cancer cells. Our current finding of an effect of XIAP^ΔRING^ expression on cancer cell anchorage-independent growth suggests that overexpression of this protein may contribute to genetic instability associated with cell cycle and checkpoint perturbations, in addition to its impact on cellular apoptosis.

## INTRODUCTION

Disequilibration between cell proliferation and apoptosis has been identified for a momentous mechanism of tumorigenesis. The X-linked inhibitor of apoptosis protein (XIAP) is a member of a larger family of proteins called IAPs (inhibitors of apoptosis), which is defined by a common characteristic ~80 amino acid domain named Baculoviral IAP Repeat (BIR) [1-3]. The BIR domains that can vary in number from one to three in different IAPs are indispensable for their anti-apoptotic activity. The RING finger domain of XIAP possesses E3 ubiquitin ligase activity, and appears to be responsible for self-degradation of these IAPs through the proteasome, in response to certain apoptotic stimuli [4]. The RING domain may not only function as negative regulator of the IAPs, for example, the RING finger of cIAP1 mediates the ubiquitination of caspases-3 and -7 [5]. The RING finger of XIAP can also mediate an interaction with Bone Morphogenetic Protein Receptor-1A (BMPR-1A), with the BIR region subsequently recruiting TAB1 (TAK1 Binding Protein 1)/TAK1 (TGF-β-activated Kinase 1) complexes to the receptor [6]. It was reported that XIAP can activate Nuclear Factor kappa B (NF-κB) through the activation of TAK1 [7], although it is still unknown whether these activities are independent of the anti-caspase and E3 ubiquitin ligase activities of XIAP. Recently, XIAP was found to inhibit cIAP-2 auto-degradation through binding with BIR2 and BIR3 domains of c-IAP2 by RING finger to enhance IκB-α phosphorylation on serines 32 and 36[8]. In this context, it appears that XIAP functions as an adapter molecule necessary for cancer cell signaling.

Despite the overall sequence and structural similarity among the IAP family members, the subcellular distribution of IAPs seems extremely variable. For example, survivin is predominantly a nuclear protein, and its expression is cell cycle dependent, peaking at G_2_-M in normal cells [9]. Moreover, high levels of nuclear c-IAP1 are predictive of poor overall survival and local recurrence-free survival in cervical squamous cell carcinomas, and are shown to be independent of prognostic factors by multivariate analysis [10]. Although XIAP is expressed mostly in the cytoplasm, and less extent in nuclear in normal cells [11], its higher nuclear expression is correlated with lower survival rate in breast carcinoma patients [12]. In addition, the biological function and mechanisms of nuclear XIAP is not explored yet.

In mammalian cells, the E2F family is composed by eight members and the diversity found in this family reflects distinct roles in the transcriptional regulation and cell function [13]. E2F1-3, forming heterodimers with DP proteins, functions primarily as transcriptional activators; in contrast, E2F4-8 acts mainly as transcriptional repressors. The transcription factor E2F family members have been well known for their ability to regulate cell cycle progression by coordinating a large group of genes involved in regulation of G_1_ to S phase transition [13]. G_1_ phase progression in mammalian cells is mediated by the activities of Cyclin D1-CDK4 (or CDK6) and Cyclin E-CDK2. At the molecular level, during G_1_/S, the Cyclin E–CDK2 complex hyperphosphorylates retinoblastoma protein (RB), and leads to the dissociation of E2F1 from RB and in turn initiates E2F-dependent transcriptional activity. It is therefore thought to be critical for normal cells to tightly regulate cyclin E activity; indeed, alteration of the E2F/Cyclin E axis is well-known to be involved in the cancer development in various types of tumor [13, 14].

In current study, we examined the effect of intracellular distribution of XIAP, the contribution of the BIR and RING domains to XIAP intracellular localization, and explored biological function of altering XIAP localization. Our findings indicate that XIAP is predominantly a cytoplasmic protein, and its nuclear localization is regulated by RING domain. We also found that overexpression of XIAP with the deletion of RING domain mainly localizes in nuclear and promotes aberrant cell division and anchorage-independent growth by binding with E2F1 in nuclear, in turn leading to E2F1 transactivation and Cyclin E induction.

## RESULTS

### The differential role of XIAP RING domain and its E3 ligase in promotion of cancer cell anchorage-independent growth and cell cycle G_1_/S phase transition

**Figure 1:**
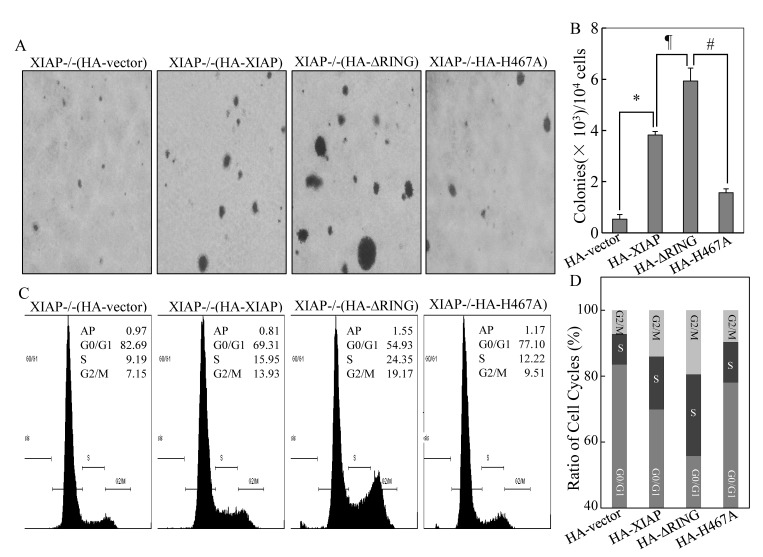
Promotion of cancer cell anchorage-independent growth and G_1_/S phase cell cycle transition by ectopic expression of XIAP^ΔRING^ (A & B) anchorage-independent cell growth of various HCT116 cell stable transfectants was evaluated in soft agar assay. The colony formation was observed and the photograph images were taken under an inverted microscope (A) and numbers of colonies were scored, and presented as colonies per 1×10^4^ seeded cells (B). The symbol (*) indicates a significant increase of colony formation in XIAP−/−(HA-XIAP) cells in comparison to that in XIAP−/−(vector) transfectant (*p*<0.05); symbol of “#” indicates a significant increase of colony formation in XIAP−/−(HA-XIAP^ΔRING^) as compared with that of XIAP−/−(HA-XIAP) transfectant; symbol of “#” shows a significant decrease of colony formation in XIAP−/−(HA-XIAP^H467A^) cells as compared with XIAP−/−(HA-XIAP^ΔRING^) transfectant. Each bar indicates the mean and standard derivation of three independent experiments. (C & D) cell cycle profile was determined by PI staining with FACS analysis in the transfectants as indicated. Representative histograms of cell cycle profiles were presented (C) and percentage of cells in the G_0_/G_1_, S, and G_2_/M phases were presented from three independent experiments (D).

In our previous study, we reported that XIAP RING domain and its E3 ligase activity are required for XIAP's upregulation of *cyclin d1* transcription and cancer cell growth [15], whereas XIAP RING domain and not its E3 ligase plays an important role in XIAP binding with RhoGDIα and inhibits RhoGDIα SUMOylation [16, 17]. To determinate whether there was a differential role of RING domain and its E3 ligase in regulation of cancer growth, we compared the capabilities of anchorage-independent growth of XIAP−/−(vector), XIAP−/−(HA-XIAP), XIAP−/−(HA-XIAP^ΔRING^), and XIAP−/−(XIAP^H467A^) in 0.33% soft agar. The results unexpectedly showed that transfection of XIAP^ΔRING^ resulted in significant increase in colony formation, while those transfected with XIAP^H467A^ showed an inhibition on colony formation as we reported before [15] (Figs. 1A & 1B). This result suggests that XIAP RING domain has an essential function in regulating cancer cell growth independent of its E3 ligase activity.

To determine the potential association of anchorage-independent growth with cell cycle alterations, we assessed the cell cycle status of the transfectants by DNA content analysis using flow cytometry. The results indicated ectopic expression of XIAP^ΔRING^, XIAP−/−(HA-XIAP^ΔRING^) exhibited accelerated progression in the G_1_/S phase of the cell cycle, resulting in decreased G_0_/G_1_ proportion (54.93% *vs* 82.69%) as compared to XIAP−/−(vector) cells, whereas the cell cycle profile of XIAP^H467A^ transfectant did not show this promotion in G_1_/S phase transition (G_0_/G_1_ proportion: 77.10% *vs* 82.69%) (Figs. 1C & 1D). Taken together, these data suggest that accelerated growth of XIAP^ΔRING^ expressing cells might be associated with promotion of G_1_/S phase cell cycle transition.

### Ectopic expression of XIAP^ΔRING^ upregulated Cyclin E expression, which was essential for XIAP^ΔRING^-mediated abnormal cancer cell growth

**Figure 2:**
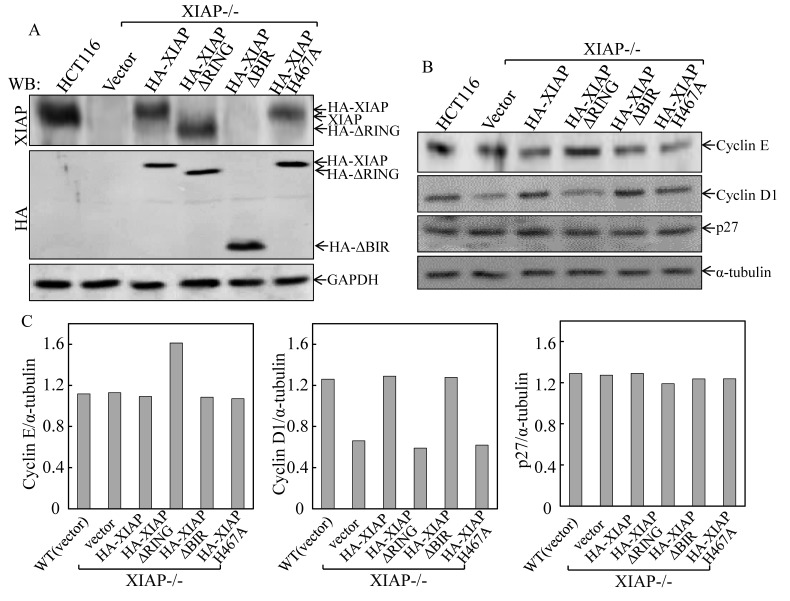
Ectopic expression of XIAP^ΔRING^ in XIAP−/− cells resulted in upregulation of Cyclin E protein expression (A) stable HCT116 XIAP−/− cell transfectants used in our study were identified by Western blot. (B & C) the cells were synchronized by incubation of cells with 0.1% FBS medium for 24h. The cells were then cultured in 2% FBS medium for 24 h and cell extracts were subjected to Western blot for determination of protein expression as indicated (B), and quantified (C).

It is known that Cyclins and CDK inhibitors are responsible for regulation of G_1_ to S transition [18]. To explore the molecular mechanisms underlying XIAP RING domain in triggering cell cycle alterations, Western blot analysis was used to identify expressions of HA-XIAP, HA-XIAP^ΔRING^, HA-XIAP^ΔBIRs^ and HA-XIAP^H467A^ in the various stable transfectants as indicated in Fig. 2A. The protein expression levels of Cyclins as well as p27 were further evaluated and compared in the transfectants. As shown in Figs. 2B and 2C, the markedly increased Cyclin E expression was only observed in XIAP−/−(HA-XIAP^ΔRING^) cells and not in any other transfectants. Consistent with our previous report [15], XIAP^−/−^(vector) and XIAP^−/−^(HA-XIAP^H467A^) cells showed a remarkable reduction of Cyclin D1 protein expression in comparison to that in XIAP−/−(HA-XIAP) cells, and p27 expression was not markedly affected. Therefore, our results demonstrated that XIAP RING domain was crucial for XIAP regulation of Cyclin E protein expression that is independent of its E3 ligase activity.

**Figure 3:**
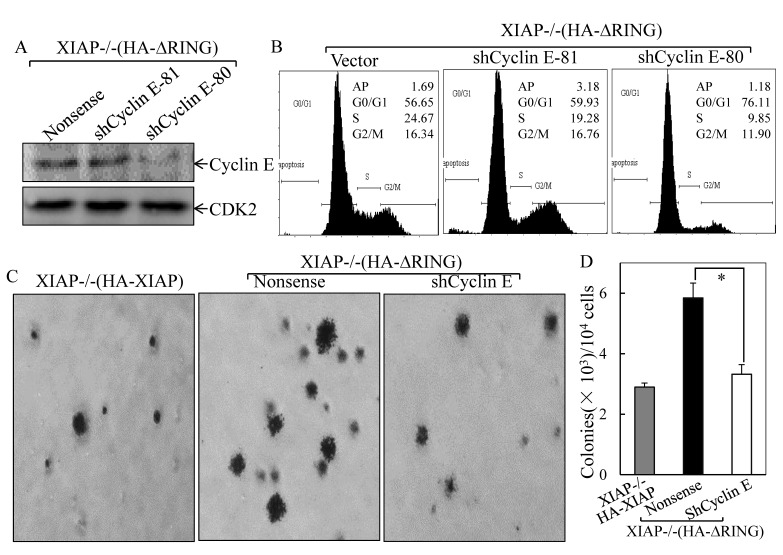
Cyclin E induction was essential for XIAP^ΔRING^ promotion of cancer cell anchorage-independent growth and G_1_/S phase cell cycle transition (A) immunoblot analysis of Cyclin E protein levels in XIAP−/−(HA-XIAP^ΔRING^) cells transfected with Cyclin E shRNA. (B) after synchronization of cells with 0.1% FBS medium, cells were cultured in 2% FBS medium for 24 h, and cells were then subjected to flow cytometry analysis. (C & D) stable transfectants as indicated were subjected to anchorage-independent growth assay in soft agar. The symbol (*) indicates a significant inhibition of colony formation by knockdown of Cyclin E expression (*p*<0.05). The colonies were expressed as mean±S.D.

To investigate the potential contribution of the increased Cyclin E expression to the XIAP^ΔRING^-mediated abnormal cancer cell growth, two independent short hairpin RNAs (shRNA) were employed to knockdown Cyclin E expression in XIAP−/−(HA-XIAP^ΔRING^) cells[19]. As shown in Fig. 3A, transfection of shCyclin E-80 dramatically reduced Cyclin E protein expression in XIAP−/−(HA-XIAP^ΔRING^) cells, whereas shCyclin E-81 was not able to show observable reduction of Cyclin E protein expression. Knockdown of Cyclin E expression by shRNA recapitulated the cell cycle transition phenotype of XIAP−/−(HA-XIAP^ΔRING^) cells (S, 24.67% vs 9.85% and G_0_/G_1_ proportion: 56.65% vs 76.11%, Figure 3B). Accordingly, the cancer cell anchorage-independent growth phenotype was also blocked by knockdown of Cyclin E expression (Fig. 3C & 3D). These results demonstrated that maintaining increased Cyclin E expression was crucial for the increased cancer cell anchorage-independent growth property of XIAP^ΔRING^ domain in XIAP−/−cells.

### XIAP^ΔRING^ regulated *cyclin e* transcription through E2F-dependent transcriptional activity

**Figure 4:**
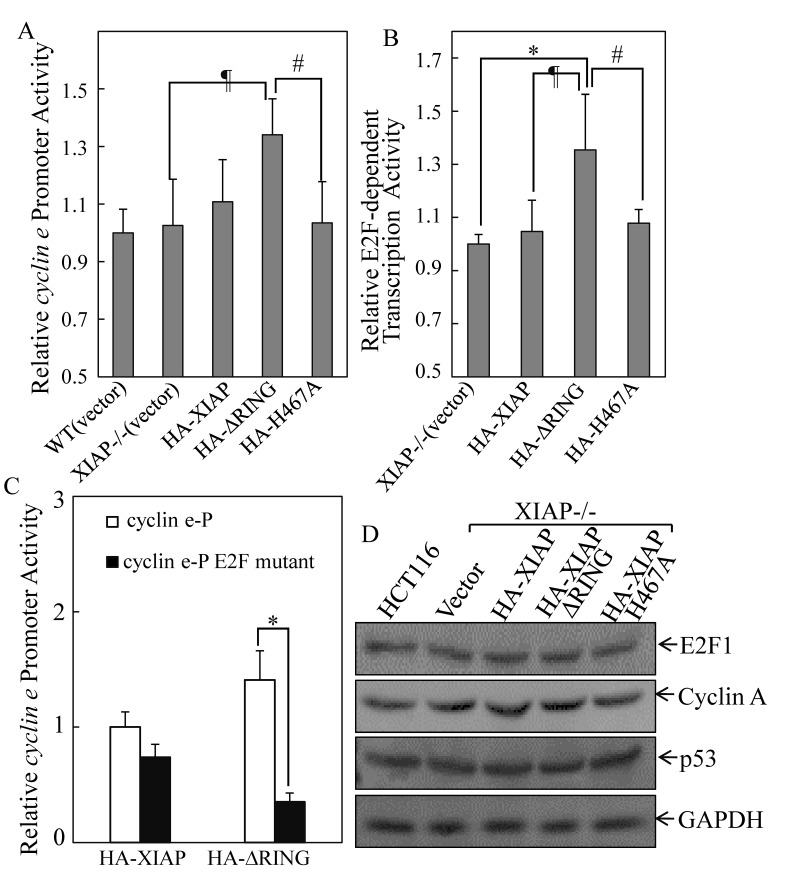
XIAP^ΔRING^ regulated *cyclin e* transcription *via* induction of E2F1 transactivation (A) the cells (1×10^4^) stably transfected with *cyclin e* promoter-driven luciferase reporter were seeded into each well of a 96-well plate. After synchronization, the cells were extracted for determination of the luciferase activity, as described in our previous studies[53]. (B) the indicated transfectants that were stably transfected with (E2F)_6_ luciferase reporter (1×10^4^) were seeded into each well of a 96-well plate and subjected to luciferase activity assay. (C) cells (1×10^4^) stably transfected with *cyclin e* promoter reporter or *cyclin e* promoter reporter with E2F binding sites mutation were seeded into each well of a 96-well plate and subjected to luciferase activity assay. (D) Western blot was performed to determine the E2F1 and E2F1 target proteins expression in the indicated transfectants.

To elucidate the molecular mechanisms underlying the regulation of Cyclin E expression by XIAP RING domain, we transfected *cyclin e* promoter-driven luciferase reporter into different XIAP transfectant cells and the *cyclin e* promoter transcriptional activity was evaluated. The results showed that *cyclin e* transcription activity was upregulated in XIAP^ΔRING^ expression cells, but not XIAP^H467A^ cells (Fig. 3A). It has been known that there are six E2F binding sites in the human *cyclin e* promoter and that the 3 upstream E2F binding sites are important for the upregulation of *cyclin e* gene transcription [20]. Thus, we examined whether XIAP^ΔRING^ enhanced E2F-dependent transcriptional activation. We transfected E2F-dependent luciferase reporter, which is driven by six tandem E2F binding sites, into XIAP−/−(HA-XIAP^ΔRING^) cells [21]. As shown in Fig. 4B, E2F-dependent transcriptional activation was significantly upregulated in XIAP−/−(HA-XIAP^ΔRING^) cells in compared with XIAP−/−(vector) cells (Fig. 3B). The increased *cyclin e* promoter transcriptional activity in XIAP−/−(HA- XIAP^RING^) cells was abolished by mutation of E2F binding site in *cyclin e* promoter-driven luciferase reporter (Fig. 3C), suggesting that the increased *cyclin e* promoter transcriptional activity XIAP^ΔRING^ domain was E2F-dependent [21]. Taken together, ectopic expression of XIAP^ΔRING^ could strongly activate E2F-dependent transcriptional activity, which conceivably underlies its ability to upregulation of Cyclin E expression, thereby contributing to acceleration of cancer cell anchorage-independent growth.

In addition to the luciferase reporter of E2F activity we also examined the steady-state expression levels of cell cycle checkpoint Cyclin A and cell apoptosis related p53, which are known to contain an E2F response element and are transcriptionally regulated by E2F1 [22, 23]. The different expression of E2F1, Cyclin A or p53 was not observed among various transfectants (Fig. 3D). These results implied that expression of XIAP^ΔRING^ accelerated anchorage-independent growth by specific upregulation of E2F- dependent Cyclin E, rather than other E2F1-targeted cell cycle and apoptosis related proteins.

### XIAP^ΔRING^ localized into nuclei in cancer cells and interacted with E2F1 through BIR domain

**Figure 5:**
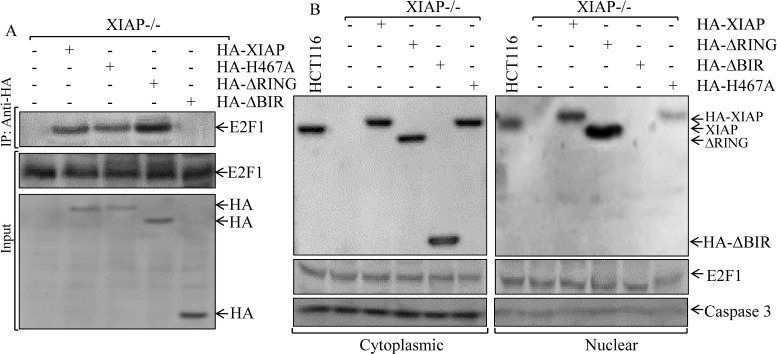
Determination of XIAP^ΔRING^ protein interaction with E2F1 protein and the distribution of XIAP protein and its various deletions between cytoplasm and nuclei (A) the cell extracts from various XIAP−/− transfectants as indicated were used for immunoprecipitation with anti-HA antibodies and pull-down protein complexes were subjected to Western blot analysis. (B) cytosolic and nuclear lysates prepared from indicated cells were analyzed by Western blot as indicated.

Given the increased E2F-dependent transcriptional activity of E2F1 in XIAP−/−(HA-XIAP^ΔRING^) cells as demonstrated above and the interaction of E2F1 and cIAP1 through BIR domain as reported in previous studies [24], we anticipated that XIAP RING domain might be able to interact with E2F1 in the intact cells. To test this notion, we carried out a co-immunoprecipitation assay as shown in Fig. 5A. Total cellular proteins from transfectants of XIAP−/−(vector), XIAP−/−(HA-XIAP), XIAP−/−(HA-XIAP^ΔRING^), XIAP−/−(HA-XIAP^H467A^) and XIAP−/−(HA-XIAP^ΔBIRs^) cells were immunoprecipitated by using anti-HA antibody-conjuncted beads. The results obtained from Western blot analysis revealed that E2F1 protein was presented in co-precipitated protein complex in XIAP−/−(HA-XIAP^ΔRING^), but not detectable in XIAP−/−(HA-XIAP^ΔBIRs^) cells (Fig. 5A), indicating that XIAP does interact with E2F1 through BIR domain in the cells. Since E2F1 acted as transcriptional factor in nucleus, we tested whether XIAP^ΔRING^ could translocate into nucleus. To establish the sub-cellular distribution of XIAP^ΔRING^, we isolated cytoplasma and nuclear fractionations in various transfectants as indicated in Fig. 5B. Examination of the fractions for XIAP^ΔRING^ sub-cellular distributions showed that XIAP^ΔRING^ mainly presented in the nuclear fraction, whereas XIAP^ΔBIRs^ and XIAP^H467A^ mainly showed in cytosolic fraction (Fig. 5B). Taken consideration of alteration of the RB-E2F axis plays an important role in cancer development in various types of tumors demonstrated in previous studies [13] and our above findings that significant increased Cyclin E protein expression, cell cycle transition promotion and anchorage-independent growth of cancer cells, we anticipated that XIAP^ΔRING^ nuclear location plays a role in the regulation of E2F transcriptional activity by its interaction with nuclear E2F1, which in turn increased E2F1-mediated transcription of its targeted gene Cyclin E.

## DISCUSSION

XIAP is an important multifunctional protein with activities and purposes reaching far beyond the mere inhibition and control of cell apoptosis [15, 17, 25]. The aim of this study was to delineate possible differences in the anchorage-independent growth regulating behavior of XIAP protein lacking of BIRs or RING domain in order to more clearly explain their contribution to the synergistic or antagonistic effects on regulating cancer cell growth. Our results revealed here that exogenous XIAP^ΔRING^ could increase anchorage-independent cancer cell growth and promotion of G_1_/S phase transition. The further study demonstrated that XIAP^ΔRING^ translocated into nuclear and this nuclear translocation was associated with the promotion of G_1_/S phase transition of cancer cell cycle by binding with E2F1 protein and subsequently resulted in E2F1 transactivation and induction of transcription of *cyclin e* in cancer cells. Our finding of the effect of XIAP^ΔRING^ on cancer cell growth and cytokinesis suggests that nuclear XIAP might contribute to genetic instability associated with cell cycle and checkpoint perturbation. Taken together with poor clinical outcome of the cancer patients with high expression of XIAP in nuclear[12], our novel findings of XIAP^ΔRING^ in nuclear location and upregulation of E2F1/Cyclin E axis provide us significant insight into understanding of nuclear XIAP in cancer patients.

XIAP^ΔRING^ expression and its translocation into nucleus resulted in its binding and interaction with E2F1 protein, by which induced in E2F transactivation and subsequently led to an E2F-dependent cyclin E induction. Cyclin E induction elicits various effects upon cell proliferation, differentiation, and survival in a manner that is highly dependent on tissue type and cell lineage[26]. The E2F-mediated Cyclin E induction could trigger a G_1_/S transition and promote an anchorage-independent growth in cancer cells. Our finding of an E2F-mediated increase of Cyclin E expression upon ectopic XIAP^ΔRING^ expression implicated XIAP as a cell cycle regulator in cancer cells. Since the cyclin E gene represents one of E2F's transcriptional targets, Cyclin E and cyclin E-CDK2 kinase activity is essential for assembly of DNA prereplication complexes and for firing of DNA replication origins[27]. These functions of cyclin E are essential when nondividing cells exit the quiescent state and resume cell proliferation but may be redundant with the activities of other cell cycle regulators in continuously proliferating cells [28]. It was reported that overexpression of cyclin E2, a subtype of E-cyclins, also induced genomic instability by a typical mechanism[29]. XIAP^ΔRING^ expression and its translocation into nucleus resulted in its binding and interaction with E2F1 protein, by which induced in E2F transactivation and subsequently led to an E2F-dependent cyclin E overexpression. A cyclin E transgene that overexpresses cyclin E induces breast carcinomas in mice, and ectopic cyclin E overexpression both induces genetic instability in cultured cells and transforms rodent fibroblasts in combination with other oncogenes [30-32]. It is therefore thought that increased cyclin E activity is a hallmark of cancer cells[14]. Many studies have shown that high cyclin E protein expression in human cancers is associated with increased tumor aggressiveness and poor patient outcome[14, 33, 34]. Our results provide significant information for understanding the increased nuclear XIAP expression in cancer cells.

In addition to tumorigenesis, dysregulated cyclin E activity also causes cell lineage-specific abnormalities, such as impaired maturation due to increased cell senescence[35]. Cell senescence is a signal transduction program leading to irreversible cell cycle arrest and is also a process that limits lifespan and the proliferation of normal cells, accompanied by a distinct set of change in the cellular phenotype [36, 37]. Cellular senescence irreversibly arrests cell growth and is a major barrier that cells must overcome in order to progress to full-blown malignancy[36]. It was demonstrated that cellular senescence is associated with hyper-mitogenic drive and failure to undergo mitosis, and cyclin E was induced in senescent cells [38]. The function of cyclin E in cell senescence was discovered by two separate groups that cyclin E-CDK2 can phosphorylate c-Myc, directing a transcriptional program that opposes oncogene-induced senescence [39, 40]. Implicit to these results is the prediction that cyclin E could mediate evasion of oncogene-induced senescence for continued growth. In the scenario of cellular senescence, enforced arrest of the cell cycle in G_0_ (e.g., caused by an increase in p21/p27) is key to senescence [36, 37]. In our experiments, ectopic expression of XIAP^ΔRING^ exhibited accelerated progression in the G_1_/S phase of the cell cycle, resulting in decreased G_0_/G_1_ proportion as compared to XIAP−/−(vector) cells. This result suggested that overexpressed Cyclin E in XIAP−/−(HA-XIAP^ΔRING^) plays a role in cell cycle regulation without cell senescence under our *in vitro* experimental condition. We realized that cell type and growth conditions play a critical role in determining phenotypes associated with high cyclin E expression[41] and the *in vivo* effect of ectopic expression of XIAP^ΔRING^ and cell senescence is under investigation in our laboratory.

Our results prompt several questions that we are investigating in ongoing work. For one, the observed translocation of XIAP^ΔRING^ into the nucleus can be brought into the account in relation to its interaction with XAF1, a cellular inhibitory factor that binds and negatively regulates the caspase-mediated XIAP cleavage [11]. XAF1 directly interacts with XIAP and antagonizes its inhibitory function on caspase activity. The nuclear localization of XIAP-XAF1 resulted in the dominance of the proapoptotic caspases as evident by the presence of decreased levels of caspases 3 and 9 in the XIAP immunoprecipitates, increased caspase 3 activity and cleaved caspase 3[11]. Taking this consideration, we hypothesize that XAF1 may be involved in regulation of XIAP^ΔRING^ translocation from cytoplasma into nuclei. The clarification of XAF1 in XIAP^ΔRING^ translocation is currently undergoing in our laboratory.

Avoidance of apoptosis is critical in the development and progression of cancer [42]. XIAP possesses three BIR domains and a carboxy-terminal RING domain. It is known that XIAP prevents caspases 9 and 3 activation through its BIR3 and the BIR2 domain, respectively, and Smac/DIABLO relieves this caspase inhibitory activity of XIAP by binding to the BIR3 domain [43, 44]. XIAP cleavage is one of the main mechanisms by which cell death programs overcome the antiapoptotic barrier posed by XIAP [45, 46], and one mechanism is a caspase-dependent cleavage of XIAP [45]. It has been reported that calpain induces a cleavage of XIAP into various fragments, by which inactivates XIAP inhibition of caspases [46]. The results from current studies demonstrated that XIAP^ΔRING^ was mainly located in the nucleus, rather than cytoplasm. We found that ectopic expression of XIAP^ΔRING^ in nuclear led to promotion of G_1_/S cell cycle transition and anchorage-independent growth. This raised the possibility that XIAP could positively impact cancer cell growth. XIAP protein was originally identified as modulators of caspase function to prevent apoptosis, and it is now clear that multi-faceted XIAP protein performed a much more diverse range of functions than first predicted.

The protein subcellular location has been reported to regulation of cellular biological function in many previous studies [47, 48]. For example, nuclear localization and export of cIAP1 are particularly interesting because cIAP1 has thus far been described to function in regulating the cytosolic activities of caspases and Smac [49]. Nowak D et al. demonstrated for the first time that XIAP translocation from the cytosolic compartment into the nucleus is observed upon treatment of cells with cytotoxic drugs [50]. This result implies a new unknown function of XIAP in nucleus in the context of drug-induced apoptosis. It has also been reported that XIAP nuclear labeling was a sign of unfavorable prognosis in breast invasive ductal carcinoma [12]. Our results demonstrated here that XIAP^ΔRING^ expressing cells exhibited a significant acceleration of G_1_/S phase transition and cancer cell anchorage-independent growth. Therefore, the presented study predominately focused on the potential regulatory role of XIAP RING domain. Since antisense oligonucleotides directed against XIAP are currently being evaluated in clinical trials and RING domain also is potent target of these agents [51], the results obtained from the current studies lead us to take a special consideration whether these drugs are able to target XIAP that is located in nucleus of cancer cells.

In summary, we demonstrated here that cancer cells transfected XIAP deletion of RING domain led to *cyclin e* transcription and protein expression *via* binding with and modulation of the E2F1 transactivation. We also showed that XIAP^ΔRING^ could translocate to the nuclear and the nuclear localization of XIAP is dependent on the BIR domains, whereas RING domain provides an inhibitory effect on XIAP translocation. More importantly, we identified that nuclear-translocated XIAP^ΔRING^ significantly promotes G_1_/S phase transition and anchorage-independent growth of cancer cells. Our results provide a significant insight into understanding of biological significance of the increased nuclear XIAP expression in cancer cells and poor prognosis of clinical patients with nuclear XIAP overexpression. In addition, the current studies also provide very useful information for new drug design, which can target nuclear XIAP in cancer cells. Determining the significance of our findings to mammary tumor biology is another focus of our ongoing studies.

## MATERIALS AND METHODS

### Cell culture and reagents

Human colon cancer cell lines HCT116 wild-type (WT) and HCT116 XIAP−/− cells, as well as their transfectants, including HCT116 WT(vector), XIAP−/−(vector), XIAP−/−(HA-XIAP), XIAP−/−(HA-XIAP^ΔBIRs^) and XIAP−/−(HA-XIAP^ΔRING^), were established in our previously published studies [15-17, 52]. The HCT116 cells and transfectants were cultured in McCoy's 5A medium (Invitrogen, Carlsbad, CA) supplemented with 10% FBS and penicillin/streptomycin. All cells were maintained in a humidified incubator at 37°C, with a 5% CO_2_ humidified atmosphere. Antibodies for immunoblotting were obtained from the following sources: XIAP polyclonal antibody (BD PharMingen, San Diego, CA); XIAP monoclonal antibody, E2F1, CDK2 and GAPDH (Cell Signaling Technology Inc., Beverly, MA); Cyclin D1, Cyclin E and CDK4 (Santa Cruz Biotechnology, Santa Cruz, CA); p27 (Abcam, Cambridge, MA); HA (Covance Inc., Princeton, NJ).

### Plasmids and cell transfection

The constructs of *cyclin e* promoter, E2F mutant *cyclin e* promoter and 6×E2F luciferase reporters were gifts from Dr. Daniel S. Peeper, the Netheorlands Cancer Institute [21]. Human Cyclin E specific shRNA constructs were gifts from Dr. Hanfei Ding, Georgia Health Sciences University[19]. Transfections were done using PolyJet^TM^ DNA In Vitro Transfection Reagent (SignaGen Laboratories, Gaithersburg, MD) and Opti-MEM^®^ reduced serum medium (Invitrogen, Carlsbad, CA). For stable transfection, cultures were subjected to blasticidin selection. These stable transfectants were cultured in the selected antibiotic-free medium for at least two passages before utilization for experiments.

### Anchorage-independent growth assay

Anchorage-independent growth ability was determined in soft agar, as described in our previous studies [53]. Briefly, 3 ml of 0.5% agar in basal modified Eagle's medium supplemented with 10% FBS was layered onto each well of 6-well tissue culture plates. Cells (1×10^4^ cells) suspended in 1 ml normal medium were mixed with 2 ml of 0.5% agar-basal modified Eagle's medium supplemented with 10% FBS, and 1 ml of mixture was added into each well over top of the 0.5% agar layer. Plates were incubated at 37°C in 5% CO_2_ for 2 to 3 weeks, and the colonies with more than 32 cells of each were scored and presented as colonies/10^4^ cells.

### Flow Cytometry Assay

Cell cycle analysis was performed as described previously [15]. Cells were harvested by trypsinization and washed once with PBS. The cells were then resuspended in 0.3 ml PBS (pH 7.4), and fixed by addition of ice-cold 80% ethanol. Fixation proceeded for at least overnight at -20°C. Fixed cells were centrifuged, resuspended in propidium iodide solution (50 mg/ml propidium iodide, 10 mg/ml RNase A and 0.1% Triton X-100 in PBS) (Sigma Chemical, St.Louis, MO) for at least 1 h at 4°C. The DNA content was determined by Flow Cytometry using the Epics XL FACS (Beckman Coulter, Miami, FL) and EXPO 32 software [54].

### Western Blot

Cells were harvested and washed with PBS. Cell pellets were resuspended and incubated with lysis buffer (10 mM Tris-HCl, pH 7.4, 1% SDS, and 1 mM Na_3_VO_4_). Equal amounts of protein lysate were subjected to sodium dodecylsulphate polyacrylamide gel electrophoresis and electrophoretically transferred to PVDF membranes. Primary antibody and secondary alkaline phosphatase-conjugated antibody incubations were performed in 5% non-fat milk and 5% BSA solution in PBS-T (PBS with 0.1% Tween 20). Blots were detected by the enhanced chemifluorescence Western blot system (Model Storm 860, Molecular Dynamics) as described in our previous publications [55, 56]. Quantitation was analyzed densitometrically using AtlasImage software (version 2.01, Clontech Laboratories, Sunnyvale, CA).

### Luciferase Reporter Assay

HCT116 cells were transfected with *cyclin e* promoter-luciferase, E2F mutant *cyclin e* promoter-luciferase and 6×E2F luciferase reporters in combination with the pRL-TK vector (Promega, Madison, WI). The transfectants were seeded into 96-well plates. After the cell density reached 70-80%, cells were treated as indicated in the figure legends, and were then extracted with luciferase assay lysis buffer (Promega, Madison, WI). The luciferase activity was determined with the Dual-Luciferase Reporter Assay System according to the manufacturer's instructions as described [57, 58].

### Subcellular Fractionation

Preparation of subcellular fractions was performed as previously described [53]. The subcellular fractions were extracted according to the protocol of the Nuclear/Cytosol Fractionation Kit (BioVision Technologies, Mountain View, CA). Equal amount of protein in each sample was determined using a protein quantification assay kit (Bio-Rad, Richmond, CA). Nuclear extracts were stored at -80°C until they were used.

### Immunoprecipitation

For immunoprecipitational experiments, cells transfected with the indicated plasmids were lysed in cell lysis buffer (1% Triton X-100, 150 mM NaCl, 10 mM Tris, pH 7.4, 1 mM EDTA, 1 mM EGTA, 0.2 mM Na_3_VO_4_, 0.5% Nonidet P-40, and complete protein mixture inhibitors from Roche Applied Science) on ice. Any insoluble material was removed by centrifugation at 16,000×g for 20 minutes at 4°C. Immunoprecipitation was carried out by incubation of cell lyses with anti-HA antibody-conjugated agarose beads. After an overnight incubation, beads were washed three times with immunoprecipitation lysis buffer, and bound proteins were subjected to Western blot assay [17].

### Statistical analysis

Student's t test was utilized to determine the significance of differences between different groups. The differences were considered to be significant at *P*<0.05.

## Abbreviations used in this paper

BIR: The Baculovirus IAP repeat
BMPR-1A: Bone Morphogenetic Protein Receptor-1A
CDK: Cyclin-dependent kinase
IAP: inhibitors of apoptosis protein
NF-κB: Nuclear factor Factor kappa B
RB: retinoblastoma protein
RING: really interesting new gene
TAB1: TAK1 Binding Protein 1
TAK1: TGF-β-activated Kinase 1
XIAP: X-linked inhibitor of apoptosis protein.
